# Primary microcephaly: do all roads lead to Rome?

**DOI:** 10.1016/j.tig.2009.09.011

**Published:** 2009-11

**Authors:** Gemma K. Thornton, C. Geoffrey Woods

**Affiliations:** Department of Medical Genetics, Cambridge Institute for Medical Research, Wellcome/MRC Building, Addenbrookes Hospital, Cambridge, UK, CB2 0XY

## Abstract

The relatively large brain and expanded cerebral cortex of humans is unusual in the animal kingdom and is thought to have promoted our adaptability and success as a species. One approach for investigating neurogenesis is the study of autosomal recessive primary microcephaly (MCPH), in which prenatal brain growth is significantly reduced without an effect on brain structure. To date, eight MCPH loci and five genes have been identified. Unexpectedly, all MCPH proteins are ubiquitous and localise to centrosomes for at least part of the cell cycle. Here, we focus on recent functional studies of MCPH proteins that reveal the centrosome as a final integration point for many regulatory pathways affecting prenatal neurogenesis in mammals.

## The genetics of MCPH

The initial definition of autosomal recessive primary microcephaly (MCPH; see Glossary) has proved useful to both clinicians and researchers: an individual with a small but structurally normal brain, a mild-to-moderate mental retardation but otherwise normal in appearance, health and neurological functioning [1,2]. As such, MCPH can be considered a ‘model’ disease to find genes that have essential and non-redundant functions in prenatal neurogenesis. The incidence is ∼1 in 10 000 in consanguineous populations, less in non-consanguineous populations.

The cerebral cortex is particularly reduced in size in this disorder, leading to an apparently ‘simplified gyral pattern’ as mantle thickness is preserved but surface area is reduced [3,4]. Magnetic resonance imaging studies of an affected family having prenatal diagnosis have shown the frontal lobes of the cerebral cortex to be particularly affected [4,5].

There are at least eight MCPH loci, and the genes underlying five of these have been identified (Table 1). The most recent MCPH gene to be identified was the MCPH7 gene *SIL/STIL,* from four consanguineous Indian families [6]. Mutations in *SIL* are a rare cause of MCPH, as are mutations in the genes encoding cyclin-dependent kinase 5 regulatory associated protein 2 (*CDK5RAP2*) and centromere protein J (*CENPJ*), as no further cases were found in a cohort of 100 Pakistani MCPH families [6]. Mutations in the abnormal spindle-like microcephaly associated gene (*ASPM*) cause 50% of MCPH, irrespective of ethnic background [2,7–9], whereas *MCPH2* mutations account for 10% of cases. Although the MCPH2 locus was discovered in 1999, the gene has eluded discovery so far despite extensive sequencing of the locus, suggesting that the mutational mechanism is unusual and not detectable by conventional exonic sequencing. A third of MCPH cases have not been linked to known loci, so further genes await discovery. All the MCPH proteins identified are ubiquitously expressed and have a centrosomal association for at least part of the cell cycle; suggesting knowledge of a centrosomal association might provide guidance in the selection of candidate genes.

Almost all mutations that cause MCPH introduce a premature stop codon, predicted to cause nonsense-mediated decay [2,9]. Whether these lead to functional nulls (no protein produced), or a significant reduction in protein levels is as yet undetermined. However, two missense mutations have been reported; one in *CENPJ* and one in *ASPM*, but their clinical significance remains unclear in lieu of functional studies [7]. This is particularly important when interpreting *ASPM* mutations as it is a large gene containing many, often rare, non-synonymous SNPs [2]. Therefore, any missense mutation in *ASPM* should be regarded as non-pathogenic until proven otherwise [2].

Microcephalic osteodysplastic primordial dwarfism - type Majewski II (MOPD-II) has an interesting phenotypic overlap with MCPH and Seckel syndrome. A disorder characterised by prenatal onset proportionate short stature, primary microcephaly and a distinctive facies associated with severe developmental delay. MOPD-II results in a marked microcephaly of similar severity to MCPH but with proportionate short stature, which is not seen in MCPH (there is some speculation that *Homo floresiensis* is a case of MOPD-II; Box 1). In further contrast to MCPH, individuals affected with MOPD-II have cognitive abilities within the normal range. The MOPD-II gene has recently been identified as pericentrin (*PCNT*) [10–12], which encodes a key centrosomal protein, a further indicator that centrosomal proteins and/or centrosomes act as key organisers of early neurogenesis.

## What do MCPH proteins do?

Recent studies of the functions of MCPH proteins have produced some interesting results. Whereas previous studies indicating roles for ASPM in spindle organisation, CDK5RAP2 and CENPJ in transcriptional regulation and microtubule dynamics, or MCPH1 in DNA damage have been expanded upon, new roles and connections have been uncovered. Recent reports may begin to explain how these proteins influence neurogenesis, including a possible functional relationship between PCNT and MCPH1, and there is evidence to suggest that MCPH proteins have a role in the progression of other diseases (Box 2).

### MCPH1: a protein of two halves

The loss of MCPH1 in experimental systems including fruit flies (*Drosophila melanogaster*), chicken cell lines or human patient cell lines, results in a range of phenotypes, including reduced protein recruitment to centrosomes, formation of abnormal spindles, missegregation of DNA, aberrant cytokinesis, changes in cell-cycle progression and defects in DNA damage repair [13–25]. But how are these phenotypes interconnected? Does MCPH1 have one or many roles? What is the contribution of the three BRCA1 C Terminal (BRCT) domains of MCPH1? Recent research has contributed further understanding of the multiple pathways and processes requiring MCPH1.

MCPH1 contains three BRCT domains (BRCT1–3), which control various aspects of its function. Current research suggests the N-terminal BRCT domain (BRCT1) of MCPH1 predominantly regulates its centrosome and cell-cycle regulatory functions. In chicken DT-40 cells, MCPH1 localisation at the centrosome after irradiation requires BRCT1, independent of DNA damage signalling pathways. Centrosomal localisation persists in the absence of DNA damage throughout the cell cycle, although this is not completely dependent on BRCT1 [17].

A study of patient lymphoblastoid cells found that centrosomal localisation of PCNT is reduced if MCPH1 is lost, whereas loss of either protein reduces recruitment of γ-tubulin. MCPH1 and PCNT together recruit Chk1 kinase to the centrosome and, thus, are required for maintaining the inhibitory phosphorylations of Cdk1 kinase on Tyrosine15 and Cdc25B phosphatase on Serine230, which together prevent mitotic entry. Loss of MCPH1 reduces inhibition, promoting a premature entry into mitosis, which is manifested as premature chromosome condensation (PCC) [22,25]. Reduced phosphorylation of Tyrosine15 of Cdk1 was also seen in one study of *Drosophila melanogaster mcph1* mutants [13]. This might explain why, although MCPH1 and Condensin II directly interact, expression of the interacting region alone could not reverse PCC, whereas expression of BRCT1 could [16]. Therefore, the loss of mitotic entry inhibition might provide the signal prematurely activating Condensin II and promoting PCC, rather than the loss of MCPH1 and Condensin II association. The requirement for both MCPH1 and PCNT for proper Chk1 recruitment might explain the similarities observed between MCPH and MOPD-II phenotypes [11,12,22].

The C-terminal domains, BRCT2 and 3, largely coordinate the DNA damage response, and it appears that MCPH1 is involved at multiple stages in response to a range of DNA-damaging insults [15,17]. MCPH1 recruitment to DNA damage foci is rapid and depends on BRCT2 and 3 binding specifically to phosphorylated γH2AX, the first protein recruited [15]. MCPH1 recruitment precedes other DNA-damage mediators; lying upstream in both ataxia telangiectasia mutated/ ATM and Rad3-related (ATM/ATR) pathways, it is required for the recruitment of Rad51, BRCA2, 53BP1, mediator of DNA-damage checkpoint 1 (MDC1), phospho-ATM, phospho-NBS1, replication protein A (RPA) and Rad17 [15,16,18,23]. Similarly, MCPH1 is recruited to telomeres, where it regulates the response to DNA damage and telomere dysfunction foci formation [21].

There is evidence to suggest that MCPH1 also regulates the transcription of DNA-damage genes. An interaction between MCPH1 and the E2F transcription factor 1 (E2F1) in unstimulated cells is enhanced after application of a radiomimetic drug. Binding required the BRCT2 and 3 domains of MCPH1; and MCPH1 and E2F1 were shown to co-occupy the promotors of BRCA2, CHK1, p73 and caspase7. The expression of DNA damage response genes topoisomerase (DNA) II binding protein 1 (*TOPBP1*), *RAD51*, and damage-specific DNA binding protein 2 (*DDB2*) was also found to be MCPH1 dependent, suggesting that a subset of E2F1 targets that are important for apoptosis are also dependent on MCPH1 regulation [20]. Finally, BRCT1 also contributes to DNA-damage repair, as it is required to interact with the switch/sucrose nonfermentable (SWI–SNF) chromatin remodelling complex and thereby regulate chromatin relaxation. This change in chromatin conformation facilitates access of DNA-damage repair proteins modulating repair efficiency and cell survival [24].

The functions of MCPH1 can therefore be subdivided into cell cycle and/or centrosome effects mediated by the N-terminal BRCT1 domain, and regulation of DNA-repair efficiency predominantly mediated through BRCT2 and BRCT3. Which of these functions is key for neurogenesis has yet to be determined, because both cell-cycle length and defective DNA damage responses have been shown to affect neurogenesis [26].

### ASPM: spindle organiser and rotational regulator

ASPM is the only MCPH protein for which a role in neurogenesis has been directly assessed. *Aspm* mRNA is expressed in the ventricular zone (VZ) of mouse neuroepithelium, particularly in progenitors undergoing proliferative divisions. Expression is high at the onset of neurogenesis and during the early stages, decreasing progressively as neurogenesis proceeds. Aspm protein localises to mitotic spindle poles with, but not overlapping, γ-tubulin, consistent with previous data [1,27]. A mouse knock-in line where GFP is expressed only in neurogenic progenitors demonstrated that levels of Aspm were reduced in neurogenic compared with proliferative progenitors.

However, RNAi knockdown of *Aspm* in the neuroepithelium did not affect cell-cycle progression or block mitosis, and centrosome detachment only occurred during telophase. Instead, loss of Aspm resulted in an increased deviation of the cleavage plane in proliferative neuroepithelial progenitors, causing almost 50% of them to bypass the apical membrane and resulting in unequal inheritance of this domain by the daughter cells (Box 3). As a result of this bypass, an increase in neuron-like progeny was observed. This suggests that loss of Aspm disrupts cleavage plane alignment, causing an increase in non-neuroepithelial progeny and depleting the progenitor pool prematurely [27].

Interestingly, the nematode *Caenorhabditis elegans* orthologue ASPM-1 has a similar role regulating meiotic spindle rotation, but is dispensable during early mitotic divisions in contrast to other organisms. Calmodulin (CMD-1) recruits ASPM-1, and together they recruit LIN-5, forming a complex at meiotic and mitotic spindle poles. RNAi knockdown of either *aspm-1* or *cmd-1* caused defects in meiosis I and II, affecting the coordination of chromosome segregation and spindle positioning. Spindle repositioning to the cell cortex was independent of the ASPM-1–CMD-1–LIN-5 complex. Once repositioned, the spindle normally rotates by 90^o^; however, rotation was defective in *aspm-1*, *cmd-1* or *lin-5* knockdowns. Further experiments suggest LIN-5–ASPM-1–CMD-1 regulates spindle positioning through dynein recruitment. However, disorganised spindles with unfocused spindle poles were observed only after *aspm-1* depletion, suggesting that ASPM-1 alone is required for spindle organisation [28].

In general, *ASPM* expression has been linked to proliferation and is highest in progenitor cells. Mouse *Aspm* is downregulated upon neurosphere differentiation, and knockdown of *Aspm* reduced both the self-renewal capacity and proliferation of neurospheres upon re-culture. This suggests that ASPM is important for maintaining the proliferative capacity of progenitors [29]. In agreement with this, *ASPM* expression is high in fetal tissues and cancer cell lines, but low in adult tissues [30]. ASPM might also contribute to adult neurogenesis, as stimulating proliferation of neural precursors in adult rat hippocampus induced *Aspm* expression [31].

ASPM is therefore highly expressed in progenitor cells and regulates spindle organisation and positioning. In mouse neurogenesis, this appears to be directly linked to fate determination for neuroepithelial (NE) progenitors, and a similar premature depletion of the progenitor pool could explain the reduced numbers of neurons in patients with MCPH.

### CENPJ: how does your centriole grow?

CENPJ, also known as CPAP, is a centriolar protein. Loss of the CENPJ orthologue *dSAS-4* in *Drosophila* leads to the loss of centrioles; although knockout flies survive until adulthood, their coordination is poor and viability is also low [32]. Further study of a *sas4* knockout mutant and a dominant negative demonstrated a requirement for dSas-4 in spermatogenesis, where loss of centrioles results in abnormal spindle formation and DNA segregation defects [33]. A similar requirement for dSas-4 in early embryogenesis has also been demonstrated [34].

**Figure 1:**
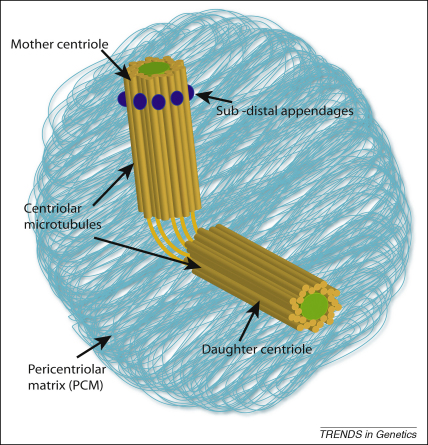
The structure of the centrosome. The centrosome is a large complex structure. At its centre sit two centrioles, which are orientated perpendicularly to each other and are linked (orange lines). One centriole, termed the ‘mother’ centriole, is older and fully mature, whereas the other is called the ‘daughter’ centriole. The mother can be distinguished from the daughter by the presence of the sub-distal appendages (purple). Each centriole takes 1.5 cell cycles to reach maturation. The centrioles are barrel-like in appearance and are surrounded by a ninefold symmetrical arrangement of triplet microtubules (orange rods). The centriole pair accumulate numerous other proteins to form the PCM. The centrosome is one of the main microtubule organising centres in the cell, and is also implicated in coordinating many pathways.

Dynamic studies of SAS-4 in *C. elegans* have also been informative in delineating function in centriole biogenesis. A SAS-4::GFP fusion protein was found to localise to centrioles in S phase and weakly to the surrounding pericentriolar matrix (PCM; Figure 1), although PCM recruitment increased in late prophase, giving a biphasic appearance to SAS-4 dynamics. Centriolar recruitment was coincident with SAS-6 rather than following it, as had been suggested in previous studies based on analysing fixed cells [35]. A dynamic equilibrium exchanging SAS-4 between the cytoplasmic pool and the centriole and/or PCM was observed that persisted until the recruitment of microtubules to the centriole at late prophase, whereupon SAS-4 incorporation appeared to be stabilised. This PCM recruitment and subsequent stabilisation required γ-tubulin and microtubules [36].

What is the function of CENPJ in human cells? Previous work had identified both a microtubule-binding domain (MBD) and a microtubule-destabilising domain (MDD) within CENPJ [37]. The MDD region has been refined to aa311-422 [38,39] and is conserved in *Drosophila* dSAS-4. It was shown that the MDD domain binds to tubulin with a stoichiometry of 1:1 sequestering it in an unpolymerisable complex, and forming a tight association that interferes with the longitudinal intermolecular interactions of β-tubulin [39].

**Figure 2:**
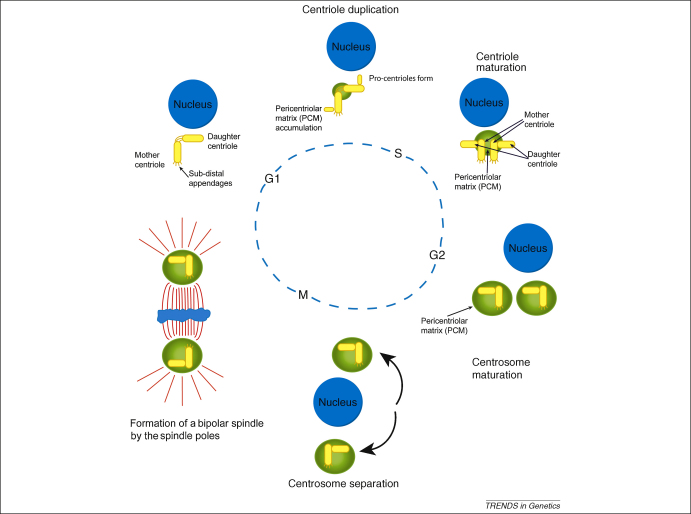
The centrosome division cycle. Centrosome duplication and maturation are linked to cell-cycle progression. In early G1, post division, the cell contains one centriole pair consisting of a mature mother centriole, which has sub-distal appendages, and an immature daughter centriole, connected to each other by a linker. During G1, pro-centrioles form perpendicularly to both the mother and daughter centrioles and continue to lengthen as G1 progresses. Around S-phase, the original daughter centriole reaches maturation, acquiring sub-distal appendages, and the link between the original centriole pair is broken. PCM proteins begin to accumulate during centriole duplication, forming two centrosomes each containing a centriole pair. This accumulation continues through the G2 phase as centrosomes mature. The final steps in maturation are the addition of centriolar microtubules around late prophase. The centrosomes, which until now have remained associated with each other, separate and move to opposing sides of the nucleus. During mitosis a bipolar spindle forms to ensure faithful DNA segregation and, at each end, there is a spindle pole each containing a centrosome. The centrosome is responsible for generating the astral microtubule array, which enables correct spindle orientation to occur. Upon cytokinesis, each daughter cell inherits a single centriole pair and the cycle begins again.

How is this role in microtubule dynamics related to cellular function? CENPJ is one of four highly conserved proteins required for centriole biogenesis (Figure 2). Two studies of overexpression of CENPJ showed formation of elongated ‘threads’ beginning at S-phase, which rapidly elongated during the G2 and M phases. The threads contained acetylated tubulin and polyglutamylated tubulin (both indicative of stabilised microtubules), and centriolar proteins, including Cep135, centrins and centrobin [40,41]. Threads occurred at both the centriole and procentriole; and although proximal centriolar structures appeared normal, the distal ends were distorted, with incomplete microtubule formation and random positioning of subdistal appendages. Despite this, they could recruit PCM, resulting in formation of extra procentrioles, increased multipolar spindle formation and defective mitoses. Interestingly, both studies also identified Cep110 as an antagonistic partner to CENPJ in regulating centriole length [40,41]. One group also observed a proteasome-mediated cell-cycle-dependent downregulation of CENPJ levels at the end of mitosis and start of G1 and identified the elements in CENPJ required for recognition by the APC/C^Cdh1^ degradation complex [41].

CENPJ is therefore a crucial regulator of centriole length during centriole biogenesis, possibly functioning in the correct recruitment of centriolar microtubules. Proteasomal degradation prevents excess accumulation in the next cell cycle promoting formation of extra centrosomes. Loss of centrioles results in deformed spindles and DNA segregation defects. Loss of CENPJ could result in an MCPH phenotype through lack of mature centrosomes, as astral microtubules would not be generated properly and spindle positioning impaired. This might lead to deviations in cleavage plane alignment similar to ASPM; however, this hypothesis needs confirmation in experimental systems.

### CDK5RAP2: the centrosome tether and PCM builder

CDK5RAP2, also known as Cep215, was previously reported to associate with the γ- tubulin ring complex (γ -TURC), involved in nucleating microtubules at the centrosome. A recent study has now shown a direct interaction between CDK5RAP2 and γ-TURC components. Overexpression of the γ-TURC binding region of CDK5RAP2 acted as a dominant negative, sequestering γ-tubulin and leading to unfocused interphase microtubules lacking a normal radial array pattern; in mitotic cells, spindle poles had fewer astral microtubules and reduced γ-tubulin accumulation. Despite this, mitosis and spindle checkpoints appeared to be unaffected [42]. Overexpression of full-length CDK5RAP2 caused protein aggregates to form both at the centrosomes and in the cytoplasm. These also accumulated increased PCNT, γ-tubulin, Cep250 and tubulins, and could nucleate microtubules without centrioles. CDK5RAP2 is centrosomal throughout the cell cycle, and immunoEM showed localisation to the PCM adhering to the centrioles and centriolar appendages [42].

An siRNA screen identified CDK5RAP2 as one of a few proteins required for centrosome cohesion [43]. Knockdown of CDK5RAP2 led to centrosome splitting, and reduced PCNT localisation at the centrosome, although other proteins tested localised normally. CDK5RAP2 localisation to the centrosome is also partially dependent on PCNT [43,44]. Interestingly, another group found that a mitosis-specific increase in both CDK5RAP2 and PCNT (along with Cep192) localisation to the centrosome was abrogated when Plk1 (polo like kinase 1) was inhibited [44].

Studies of centrosomin (cnn), the *Drosophila* homologue of CDK5RAP2, suggest that it is required for centrioles to maintain a stable connection both to accumulated PCM during centrosome maturation, and to astral microtubule arrays generated by the centrosome during mitosis. Without cnn, centrioles become displaced to the PCM periphery before losing contact and migrating randomly in the cytoplasm [45]. A screen for proteins involved in centriole duplication and centrosome maturation (Figure 2) found that disruption of cnn or P could completely suppress centrosome maturation [46]. The authors identified a Polo kinase dependent phosphorylation of cnn in mitosis (although they did not show it was direct), and a co-dependency for centrosomal localisation, similar to that seen for the respective orthologues CDK5RAP2 and Plk1 in mammals [44,46].

Centrosomin contains two motifs (cnn motifs). Loss of cnn motif 1 disrupts the ability to rescue lethality of a *cnn* knockout and centrosome separation fails, leading to increased pairs of centrosomes. Centrosomal localisation of D-TACC and γ-tubulin is reduced, while another protein, Mps1, becomes displaced to the centrosome periphery, suggesting impaired recruitment. This also suggests cnn motif 1 regulates the assembly of PCM proteins required for microtubule-organising activity at the centrosome [47]. By contrast, cnn motif 2 disruption does not affect localisation or microtubule organising activity, but causes a cleavage furrow formation defect in the early *Drosophila* embryo [48]. However, as this process is absent from mammalian development, the relevance for human studies remains unclear.

Thus, CDK5RAP2 orthologues regulate centrosome maturation, recruitment to and strengthening of the PCM at the centrioles, and might also regulate centrosome cohesion. In their absence, centrosomes fail to mature, cannot efficiently organise microtubules, and generation of astral microtubules is reduced., A similar defect in humans might lead to minor spindle positioning defects that cannot be tolerated in neuroepithelial progenitors.

### SIL: a new MCPH, familiar yet different

The MCPH7 locus and causative mutations in SIL/STIL were reported during early 2009. mRNA expression was detected in many tissues, including brain at 16 weeks of development; while *in situ* data on the Genepaint website shows subventricular neuroepithelial expression at embryonic day 14.5 (E14.5) in mouse [6] (http://www.genepaint.org).

SIL was previously identified at the site of a genomic rearrangement in a T-cell acute lymphoblastic leukaemia (hence the name – *SCL* Interrupting Locus) [49]. It is an early response gene and a target of the E2F transcription factor family; E2F1 and E2F4 have the highest affinity for the SIL promoter. *E2F1* siRNA reduced SIL expression, leading to delayed transition through mitosis [50,51]. SIL is hyperphosphorylated in mitosis in a Cdc2 (CDK1)-dependent manner, a modification that promotes interaction with peptidyl prolyl isomerase (Pin1) and seems to be required for maintenance of the spindle checkpoint. Without SIL (or hyperphosphorylation), the levels of activating phosphorylation of Cdc2 decrease, lowering the activity of Cdc2/CyclinB1 and enabling cells to escape the checkpoint block [52].

The mouse orthologue SIL is expressed in all tissues, but is highest in bone marrow, thymus and spleen. It is downregulated upon terminal differentiation in haematopoietic and erythroleukaemic mouse cell lines [53]. A mouse knockout of *SIL* is embryonic lethal at E10.5. Between E7.5 and E8.5, the knockout embryos are smaller, display pericardial swelling, midline neural tube defects, failure of neural tube closure and holoprosencephaly. A block in Sonic Hedgehog (Shh) signalling is observed, which causes a failure in left–right specification. The numbers of cells in neural folds is reduced compared with those in wild-type embryos, caused by increased apoptosis in neural folds and somites [54]. This discrepancy between human and mouse phenotypes is currently unexplained.

Cassiopeia (*csp*) is a zebrafish mutant with a loss-of-function mutation in Sil, and is embryonic lethal. Increased mitotic cells were seen, with defects including monopolar spindles, loss of polarity, misaligned chromosomes, and broadened spindle poles. γ-tubulin staining suggested that one or both spindle poles were often missing [55]. Endogenous SIL protein expression could be detected only in metaphase HeLa cells, where it localised to spindle poles in a pericentriolar manner, similar to ASPM. No expression was detected at anaphase. siRNA in HeLa cells led to an increase in cells with monopolar or unfocussed poles and disorganised spindles. In the most severe cases, centrosomes were not localised to spindle poles and dynactin recruitment was lost. The phenotype of *csp* suggests a role in spindle organisation, similar to that of ASPM/asp [55].

In summary, the data so far suggest that SIL has some similarities with ASPM, although with additional roles in spindle checkpoint regulation and Hedgehog signalling pathways. It is tempting to hypothesise that loss of SIL results in MCPH through a similar spindle pole mechanism. Patients with SIL mutations do not have obvious developmental defects in Hh signalling so this role might not be conserved.

## Concluding remarks

**Figure 3:**
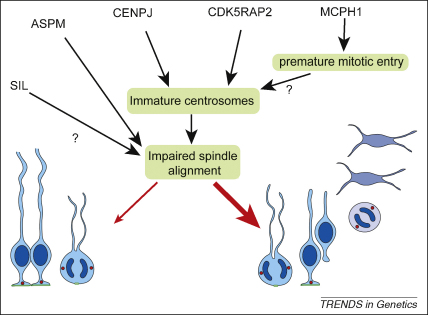
A model suggesting how loss of the MCPH proteins could affect neurogenesis. Although the MCPH proteins act in diverse pathways, these can be shown to intersect, resulting in a common mechanism affecting neuron production. Loss of MCPH1 results in a shorter G1 phase of the cell cycle through premature mitotic entry, meaning that the centrosomes have not had sufficient time to mature before the onset of division. Deficiencies in CDK5RAP2 or CENPJ directly affect centrosome maturation (in the case of CENPJ loss, centrioles are no longer able to form). Immature centrosomes accumulate less PCM, and are also less able to generate astral microtubules. This is important because astral microtubules contact the cell cortex and provide information guiding spindle orientation during division. By contrast, ASPM and SIL localise specifically to mitotic spindle poles, where ASPM directly regulates spindle positioning. We speculate that SIL has a similar role. In apical NE progenitors, spindle positioning is tightly controlled to ensure bisection of the apical plasma membrane during symmetric division. By impairing spindle orientation even mildly, loss of any MCPH protein would lead to an increase in NE cells producing neurogenic progeny upon division (wide red arrow). The proportion of symmetric divisions is concomitantly reduced (thin red arrow), depleting the progenitor pool and limiting the total number of neurons that can be generated.

How close are researchers to understanding, or identifying, a single pathway that explains how primary microcephaly proteins regulate brain growth? At first glance, the MCPH proteins operate in diverse pathways, from transcriptional regulation (MCPH1, CENPJ and CDK5RAP2), cell-cycle progression and checkpoint regulation (MCPH1, CENPJ and CDK5RAP2), centrosome maturation (CDK5RAP2 and CENPJ), DNA repair (MCPH1) to progenitor proliferation capacity (ASPM and STIL). A case can be made for all these pathways affecting neurogenesis; indeed, alterations of cell-cycle length, spindle positioning or DNA repair efficiency have been shown to reduce cortical expansion [26,27,56]. So what is the probable explanation? A direct interaction between any MCPH proteins to suggest formation of a common complex has not yet been reported. However, the conserved centrosomal association of all MCPH proteins is remarkable, and might indicate a role for the centrosome in coordinating many diverse regulatory pathways. Disruption of all the MCPH proteins can be shown to either delay centrosome maturation or to disrupt spindle orientation directly. Delayed centrosome maturation will always affect the daughter centriole and centrosome more than the matured mother centriole and centrosome, reducing the recruitment of PCM and accessory proteins, and deceasing microtubular nucleation. This yields two unequal centrosomes potentially unable to position the spindle symmetrically. Inequality of the mother and daughter centrosomes in apical neural precursor cells, during the early symmetrical cell divisions, might therefore provide a common functional link between all MCPH proteins, as illustrated in Figure 3. We believe that this is an intriguing hypothesis for future work.

Furthermore, we suggest, as have others, that the observed disease pathology is a consequence of the sensitivity of apical NE progenitor cells to perturbations, where inaccuracies tolerated in other tissues have consequences for fate specification for daughter cells upon division. In NE progenitors, spindle positioning significantly contributes to the process guiding the cleavage furrow to the apical domain. Anything that reduces the fidelity of this process will lead to a premature shift from symmetrical to asymmetric cell division, resulting in reduced neuron production and a MCPH-like phenotype. The development of suitable mammalian model systems for neurogenesis in which to disrupt and study the MCPH proteins will be crucial to enable these questions to be addressed and developed further.

## Update

While this review was in production, a paper by Wang *et al.* [78] was published showing that radial glial cells (apical progenitors, which divide asymmetrically to give one radial glial cell and one basal progenitor or neuron) partition their centrosomes asymmetrically between the progeny. The mature mother centrosome remains with the cell, which becomes a radial glial cell, while the daughter centrosome is inherited by the differentiating cell. Prevention of centrosome maturation led to loss of asymmetric partitioning, a depletion of radial glial cells and an increase in non-progenitor progeny. Although it is unclear whether the MCPH proteins affect this process, or the earlier switch in progenitors from symmetric to asymmetric divisions as we have hypothesised in this review, Wang *et al.* confirm the crucial role of centrosome maturation in affecting cell fate.

## Figures and Tables

**Figure I:**
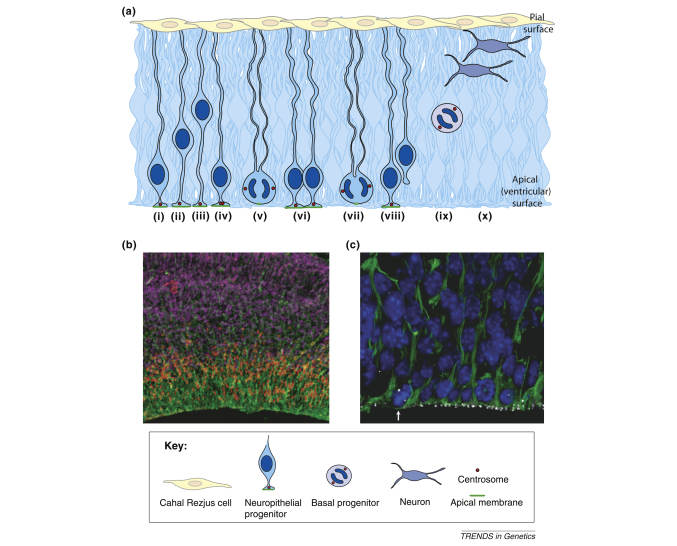
The developing mouse neuroepithelium. **(a)** The neuroepithelium and the process of cell division. Neuroepithelial cells (blue) have processes contacting the apical (ventricular) and pial (basal) surfaces. The nuclei in dark blue migrate basally during G1, cells (i,ii) undergo S phase at a basal position (iii); and migrate again apically during G2 (iv). The centrosomes (red circles) remain by the apical membrane (green). Mitosis occurs at the apical surface, where the centrosomes now form the spindle poles. Symmetrical division leads to the production of two identical neuroepithelial cells (v,vi). By contrast, asymmetrical division (vii) leads to production of one neuroepithelial cell, whereas the other daughter detaches from the membrane (viii) and becomes either a basal progenitor (ix) or a neuron. Basal progenitors (ix) lack processes and polarity and predominantly divide terminally to produce two neurons (x). **(b)** Immunofluorescence in E14 mouse neuroepithelium showing the apical progenitors and their processes (nestin, green), basal progenitors (Tbr2, red) and neurons (βIII Tubulin, purple). **(c)** Immunofluorescence in E12 mouse neuroepithelium showing the apical progenitors and their processes (nestin, green), the nuclei of the apical progenitors (DAPI, blue) and the centrosomes (γ-tubulin, white). Note the arrow pointing to the cell in metaphase.

**Table 1:** MCPH genes, proteins, localisations and functions^a^

Locus	Chromosome location	Gene	Protein	Cellular localisation	Function
***MCPH1***	8p23	***MCPH1***/*MICROCEPHALIN*/*BRIT1* (BRCT inhibitor of telomerase I)	Microcephalin/BRIT1	Nucleus/chromatin; centrosome	DNA damage repair; chromosome condensation; transcriptional regulation of DNA damage genes
***MCPH2***	19q13.12–q13.2	Not identified	Unknown	Not known	Unknown
***MCPH3***	9q33.2	***CDK5RAP2***/*CEP215* (centrosomal protein 215)	CDK5RAP2/Cep215	Centrosome throughout cell cycle; midbody at cytokinesis	Predicted role in regulating microtubule dynamics; PCM recruitment and stabilisation; centrosome maturation and cohesion
***MCPH4***	15q15–q21	Not identified	Unknown	Not known	Unknown
***MCPH5***	1q31.3	***ASPM***	ASPM	Pericentrosomal at mitotic spindle poles; midbody at cytokinesis; cytoplasmic at interphase	Spindle pole organisation and orientation
***MCPH6***	13q12.12	***CENPJ***/*CPAP* (centrosome protein 4.1 associated protein)	CENPJ/CPAP	Centrosome throughout cell cycle; midbody at cytokinesis	Centriole biogenesis and length; microtubule dynamics
***MCPH7***	1p33	***STIL*****/*****SIL***	SIL/STIL	Pericentrosomal at mitotic spindle poles	Spindle organisation; Hh/Shh signalling

a The HGNC name is highlighted in bold for each gene, although the most common alternatives are also given.

## Box 1 MCPH, evolution and the link with Homo floresiensis

There has been a clear increase in relative brain size from monkeys to apes to humans. In just 3–5 million years the human brain increased threefold in size compared with that of our closest primate relatives. This has led to a search for the genes (and the changes within those genes) responsible for this expansion. The MCPH genes were obvious candidates as mutations affect brain size exclusively, and evidence of positive Darwinian selection was found in the monkey–primate–human evolutionary tree for MCPH1, 3, 5 and 6 [1,57]. This evidence was gathered by comparing the rate of DNA base changes that do not affect protein sequence (synonymous) with those that do change an amino acid (non-synonymous) both within and between species over a defined time period. Non-synonymous changes can alter protein function. This alteration can be beneficial and subsequently either ‘selected for’, neutral, or deleterious and ‘selected against’.

Taking this approach further, when whole brain, cerebellum and cerebral cortex were analysed separately in primates, ASPM positive selection correlated only with cerebral cortex size, and appeared to be due to changes in only 16 out of the 3477 amino acids constituting ASPM [58]. Controversially, it was claimed that *ASPM* and *MCPH1* were still undergoing evolutionary change in modern humans [59–62]. Remarkably, the ‘selected’ changes were not altering either brain size or intelligence and it was hypothesized that sperm number or function was instead being affected (although *ASPM* MCPH males have fathered children). However, other groups have disputed the validity of these findings, as they could not be replicated, instead suggesting that the high variability observed in *ASPM* renders such analysis inaccurate [63,64]. Another study claimed that a *MCPH1* haplotype, present in 70% of modern humans, originated from another hominid species [65]. Such nucleotide-based analyses of *MCPH* are highly complex, however, and await careful validation by functional studies.

Finally, the remains of a dwarfed microcephalic hominid found on the Flores Islands, *Homo floresiensis,* was speculated to be a case of either MOPD-II or MCPH [11,66] rather than a new hominid species. Further analysis has now supported the original theory that *H. floresiensis* was a separate hominid species that did exist, but became extinct [67]. Despite the need for functional analyses of these changes, these reports provide exciting speculations about the development of humans as a species. As the Neanderthal genome nears completion, all the DNA changes separating modern humans from their closest hominid relative, who became extinct 30 000 years ago, will soon be known [68].

## Box 2 Are MCPH proteins implicated in other diseases?

As the MCPH proteins act in pathways affecting cell-cycle progression, proliferation and genome integrity, are there any indications of a contribution to pathogenesis in other diseases, such as cancer?

Downregulation of *ASPM* expression resulting in delayed mitotic exit was observed upon expression of a Hepatitis C virus protein that reduces p38 MAPK activation [69]. It would be interesting to investigate whether *ASPM* expression status changes during subsequent tumourigenic transformation, as *ASPM* mRNA upregulation is correlated with disease progression and poor clinical outcome in both glioblastoma and primary hepatocellular carcinoma [29,30,70,71]. Additionally, *ASPM* was one of eight genes strongly upregulated in immortalised cells and cancer cells (although not in neuroblastoma) [72].

Similarly, *SIL* mRNA is expressed in many tumours (except glioblastoma) and malignant cell lines from diverse tissues. Expression increases upon DNA synthesis induction; conversely, terminal differentiation or growth arrest leads to downregulation [51,73]. SIL relieves Gli1 inhibition through an interaction with suppressor of fused homolog (SUFU), and so promotes the transcription of target genes, including those encoding cyclin D1 and Patched; this interaction was enhanced by oncogenic K-Ras expression [74]. Conversely, inducing *SIL* knockdown in a cancer cell line resulted in a G2/M growth arrest owing to delayed Cdk1 activation and increased apoptosis, suggesting that SIL is a potential therapeutic target [50].

As for CENPJ, its expression has been detected in breast cancer cell lines [1], although there are no recent studies building on this finding; and CDK5RAP2 downregulation has been recently linked to doxorubicin and paclitaxel resistance, commonly used in combination therapy for advanced breast cancer, suggesting that CDK5RAP2 could provide a pathway for tumour cells to evade destruction [75].

In contrast to the other MCPH genes, *MCPH1* expression is downregulated in breast cancer cell lines, ovarian and prostate tumours, and is linked to increased genomic instability. Consequently, *MCPH1* has been proposed as a potential tumour suppressor [1].

What about patients with MCPH? Owing to practical difficulties, there has been little formal analysis of disease occurrence in such patients; however, anecdotal evidence suggests that the incidence of cancer is low, and possibly less than in the general population. Patients with MCPH do not appear to suffer from defective immune responses (centrosomes are involved in T-cell activation) or gut problems (a tissue that is dependent on maintaining stem cell divisions throughout life). However, although data are limited and based mainly on cell assays or mouse models, the current findings are interesting.

## Box 3 Mammalian neurogenesis: the basics

Mammalian neurogenesis is best characterised in mouse, so we will focus on this model here. The first progenitor cells in the developing brain are the apical NE progenitor cells. These are attached to the apical and pial surfaces, polarised along the apico-basal axis and form adherens junctions, which are essential for the maintenance of polarity.

NE cells adopt a ‘pseudostratified’ appearance, owing to migration of the nucleus between the ventricular (apical) surface and more basal positions (Figure Ia,b). S-phase DNA replication occurs as the nucleus reaches its most basal position (Figure Ia, iii), before returning to the apical surface to undergo mitosis. Strikingly, the centrosomes remain at the apical membrane during G1, S- and G2 phases, leading to the appearance of a ‘line’ of interphase centrosomes upon immunofluorescence imaging (Figure Ic).

The apical membrane of each NE cell encompasses a tiny but crucial fraction of the whole cellular membrane and expresses several apical proteins. Early in neurogenesis, NE cells predominantly complete cytokinesis, bisecting the apical plasma membrane. Both daughter cells receive apical membrane proteins, remain attached to their neighbours and adopt a NE cell fate. This is called a symmetrical proliferative division (Figure Ia,v, vi).

As neurogenesis progresses, progenitor divisions begin to deviate, leading to bypass of the apical membrane. This results in an asymmetrical neurogenic division (Figure Ia, vii–ix). Only one daughter receives an apical membrane and adherens junctions and remains a NE cell. The other detaches from the ventricular surface (which can take several hours) and migrates sub-ventricularly, becoming either a basal progenitor or a neuron (Figure Ia viii, ix, Figure Ib).

In contrast to NE progenitors, basal progenitors are not connected to either apical or pial surfaces and are unpolarised. These progenitors predominantly divide terminally in a symmetric neurogenic manner to produce two neurons (Figure Ia, ix, x), although a sub-population can undergo limited rounds of self expansion. The number of rounds of symmetric proliferative divisions by NE cells is thought to be crucial for the eventual numbers of neurons. A single progenitor undergoing ten rounds of asymmetric division will produce ten neurons; by contrast, if all but the last division is symmetric, ∼512 neurons can be produced.

In higher primates and humans, other progenitor populations exist that provide possibilities for a prolonged expansion phase. These are covered in detail elsewhere [76,77]. For a detailed exposition of neurogenesis, evolution and morphology, see Ref. [76].

## Glossary

Astral microtubules: the microtubule array nucleated by the centrosome during mitosis. Astral microtubules extend from the spindle poles to contact the cell cortex and function in spindle positioning.
BRCT domain: named after the protein first shown to contain this domain; it is found in proteins involved in DNA damage repair.
Centrosome: a complex multiprotein structure comprised of two centrioles surrounded by an electron-dense amorphous material, the PCM. An important regulator of many functions and the predominant site of microtubule assembly within a cell. Centrosomes co-localise with the spindle poles during mitosis.
Centriole: a multiprotein barrel-like microtubule-based structure at the core of the centrosome responsible for recruiting PCM proteins.
Cerebral cortex: also called the ‘grey matter’: the structure in the brain responsible for most information processing.
Cleavage plane: the axis along which cell division occurs.
Cohort: a group of humans with a shared diagnosis gathered and studied together to identify common features of a particular phenotype.
Consanguineous families: families in which marriages between family members (e.g. cousins) have occurred.
Hh/Shh signalling: ‘Hedgehog’ or ‘Sonic Hedgehog’ signalling pathways are involved in regulating development in several tissues.
Holoprosencephaly: a failure of the forebrain to divide to form the two cerebral hemispheres during development. Depending on the severity, this can lead to malformations of the brain and facial features, seizures and mental retardation. In very severe cases, the fetus might miscarry.
ImmunoEM: electron microscopy using antibodies conjugated to gold nanoparticles.
Locus/loci: a region in the genome that is shared by all individuals affected by a particular phenotype.
Microcephaly: the finding that an individual's head circumference is significantly below the expected mean for age and sex. The measurement must be made from the back of the head across the brow and should be the largest measurement possible. As it is the brain that pushes out on the skull and drives skull bone growth, a symmetrical microcephaly indicates a brain that is of reduced size. If the skull is asymmetric, then this usually indicates a craniosynostosis, a defect of skull bone growth and union.
Primary microcephaly: a form of microcephaly present at birth, that is not due to other pathological defects such as maternal alcoholism, diabetes or rubella.
Missense mutation: a pathogenic mutation in DNA resulting in a changed amino acid in the protein.
Neuroepithelium: the first structure formed during cortical neurogenesis.
Neurogenesis: although this term can be used to mean different aspects of brain development, for the purpose of this article, it refers specifically to the process by which neurons are generated during embryonic development.
Neurosphere: a culture derived from mouse embryonic stem cells that have formed a heterogeneous mass of cells at varying stages of differentiation along a neural lineage.
Non-synonymous SNP: a ‘single nucleotide polymorphism’ that causes a change in the amino acid encoded by the triplet that it lies within.
Pericentriolar matrix (PCM): the complex of proteins recruited to and surrounding the centrioles. Many appear to influence microtubule dynamics and other centrosome functions.
Pial surface: the pia is the outermost membrane surrounding the developing brain.
Progenitor: a cell that has some characteristics of a stem cell (i.e. it can self renew) but that has become restricted to producing differentiated cells of a particular lineage, for example neuronal cells.
Proteasome: a large protein multisubunit complex found in the cytoplasm that is responsible for the degradation of proteins to regulate their levels or to remove misfolded proteins that might be damaging to the cell. Recognition of targets for degradation is achieved through addition of many copies of a small molecule ‘tag’ called ubiquitin.
Radiomimetic: a drug, the effects of which mimic irradiation when applied to cells.
Spindle poles: where spindle microtubules converge at the two ends of the mitotic spindle. In somatic cells, the two poles of the bipolar spindle structure each contain a centrosome. The centrosomes produce astral microtubules that contact the cell cortex and are thought to provide spatial information and contribute tensile forces for chromatid separation.
Somite: ‘blocks’ of mesoderm that form sequentially either side of the extending neural tube as a mammalian embryo develops and that will later develop into skin, vertebrae and skeletal muscle. Often used by researchers instead of age to determine more precisely the exact developmental stage of an embryo (as environmental factors can influence the rate of developmental progression).
